# Comparison of whole blood and PBMC assays for T-cell functional analysis

**DOI:** 10.1186/1756-0500-6-120

**Published:** 2013-03-27

**Authors:** Anbarasu Deenadayalan, Prabhavathi Maddineni, Alamelu Raja

**Affiliations:** 1 Department of Immunology, National Institute for Research in Tuberculosis (ICMR), Chennai, Tamil Nadu 600 031, India

**Keywords:** Tuberculosis, T lymphocytes, Interferon-γ, Peripheral blood mononuclear cells, Whole blood assays

## Abstract

**Background:**

Tuberculosis remains the foremost cause of morbidity and mortality, more than any other single infectious disease in the world. Cell mediated immune response plays a crucial role in the control of tuberculosis. Therefore, measuring cell mediated immune response against the antigens is having a vital role in understanding the pathogenesis of tuberculosis, which will also help in the diagnosis of and vaccination for tuberculosis.

**Findings:**

The aim of the present study was to compare and optimize the assay conditions to measure the cell mediated immune response against *M. tuberculosis* specific antigens. Because the conventional PBMC assays (due to requirement of large volume of blood sample) are unable to screen more number of antigens within the same blood sample. So, here we have compared 6 days culture supernatants of 1:5 and 1:10 diluted blood and PBMCs from healthy laboratory volunteers, to assess the proliferative response of T lymphocytes and secreted IFN-γ levels against purified recombinant antigen of *M. tuberculosis* (MPT51, Rv3803c), crude antigens of *M. tuberculosis* (PPD) and mitogen (PHA).

**Conclusions:**

We have observed good correlation between each assay and also the mean difference of these assays did not reach the statistical significance (p > 0.05). From these results, we conclude that 1:10 diluted whole-blood cultures can be well-suited as an alternative assay to measure cytokine production and lymphocyte proliferation in comparison to the conventional PBMC assays. Moreover, 1:10 diluted blood assays require less volume of blood when compared to PBMC assays which will be useful particularly in paediatric and field studies in endemic countries, where blood volume is a limiting factor.

## Findings

### Background

Human tuberculosis (TB) is a contagious infection caused by an intracellular pathogen, *Mycobacterium tuberculosis.* Due to lack of accurate diagnostic techniques and vaccines, TB has become the second leading cause of death from an infectious disease worldwide and it was declared as ‘global emergency’ by WHO in 1993. This deadly infection leads to 8.5–9.2 million new TB cases with 1.1 million deaths annually; more over 2 billion people are latently infected with *M. tuberculosis*[1]. This situation is further complicated by the emergence of multidrug-resistant strains of bacteria and HIV co-infection.

Cell mediated immune response includes different T cells (*i.e*. CD4+, CD8+ and γ/δ T cells) and T cell derived cytokines play a pivotal role in the protection against *M. tuberculosis*[2,3]. Among the existing cytokines, IFN-γ is a key cytokine in the control of *M. tuberculosis* infection, and was confirmed by using IFN-γ knockout mice, which were more susceptible to virulent *M. tuberculosis*[4]. Individuals defective in genes for IFN-γ or the IFN-γ receptor are prone to serious mycobacterial infections including *M. tuberculosis*[5]. IFN-γ is predominantly secreted by T cells, as well as Natural Killer (NK) cells which play an important role in macrophage activation which in turn will contain and kill intracellular mycobacteria [6], control mycobacteria replication and granuloma formation both in humans and mice [7].

The ability of T cells to proliferate in response to antigen has been used as an indicator for the presence of antigen-specific T cells. Thus, measuring T cell functions in terms of lymphocyte proliferation and quantification of IFN-γ, *in vitro,* against *M. tuberculosis* specific antigens are useful immunological markers, particularly phase I and II vaccine trials and the diagnosis of tuberculosis [8].

Two different assay methods exist to analyze the T-lymphocyte functions, namely Peripheral Blood Mononuclear Cell (PBMC) assays and Whole Blood (WB) assays. The conventional PBMC assays require large volume of blood samples when compared to WB assays. Thus, WB assays gain importance in screening of large number of antigens with the same blood sample; and paediatric and field studies. So, we compared WB assay with PBMC assay to know whether WB assays can be used as an alternative or not.

In this study, we compared the lymphocyte proliferation and IFN-γ levels in 1:5 and 1:10 diluted blood and PBMC against MPT51 recombinant antigen of *M. tuberculosis,* Purified Protein Derivative (PPD) and Phytohemagglutinin (PHA).

## Methods

### Study subjects

The study was approved by the Institutional Ethics committee of National Institute for Research in Tuberculosis (NIRT) and written consent was obtained from the volunteers before recruitment into the study. Eight healthy laboratory volunteers were recruited in the present study from NIRT (formerly Tuberculosis Research Centre), Chennai, India. These individuals had no history of tuberculosis on the basis of personal history; physical examination, chest x-ray, and negative acid fast bacilli sputum smear microscopy. All the study subjects were TST positive. All participants aged from 25 to 45 years. All subjects were HIV negative as determined by Tridot (J.Mitra & co, India) and Retroquic (Qualprodiagnostics, India) assays with serum.

### Antigens and mitogens

*M. tuberculosis* PPD was obtained from Statens Serum Institute, PHA was purchased from Sigma Chemical Company, MO, USA and MPT51 recombinant protein was cloned, over expressed and purified as described previously [9].

### Lymphocyte proliferation assay

Briefly, 10 ml of heparinized venous blood was drawn from each study subject. For WB assay, 1:5 and 1:10 dilutions were made with sterile RPMI 1640 medium (Sigma Chemical Company, MO, USA), supplemented with penicillin (100 IU/ml), streptomycin (0.1 mg/ml), L-glutamine (0.29 gm/l) and amphotericin B (5 mg/ml) and was seeded in 96-well flat bottom plates at 200 μl/well.

PBMC were isolated by Ficoll-Hypaque density centrifugation. A total of 2x10^5^cells/well were cultivated in complete culture medium, supplemented with 10% Human AB serum. Cultures were stimulated either with MPT51 recombinant protein of *M. tuberculosis* (5 μg/ml), or PHA (5 μg/ml) or PPD (5 μg/ml). Cells cultured under similar conditions without any stimulation served as the negative control. The cultures were set up in triplicates and incubated for 6 days at 37°C in 5%CO_2_ atmosphere.

Sixteen hours before termination of cultures, 1 μCi of tritiated (^3^H) thymidine (Board of Radiation and Isotope Technology, MA, USA) was added to each well. The cells were then harvested onto glass fiber filters on a cell harvester and allowed to dry overnight. 2ml of scintillation fluid (0.05 mg/ml POPOP and 4 mg/ml PPO in lit. of toluene) was added to each tube containing the dried filter discs and counted by using a liquid scintillation beta counter.

The proliferation was measured as uptake of tritiated thymidine by cells and expressed as stimulation index (SI) which was calculated as

$$\text{Stimulation}\;\text{Index}\;\left(\text{SI}\right)=\frac{\text{Mean}\;\text{counts}\;\text{per}\;\text{minute}\;\text{with}\;\text{antigen}}{\text{Mean}\;\text{counts}\;\text{per}\;\text{minute}\;\text{without}\;\text{antigen}}$$

### Interferon-γ measurement

For quantification of IFN-γ, in all 1:5 and 1:10 diluted blood and PBMC cell-free culture supernatants from lymphocyte proliferation assay were harvested after 6 days of *in vitro* stimulation with or without antigen stimuli and stored at −80°C until assayed. IFN-γ production was determined by standard ELISA technique using commercially available BD opt-EIA Kit (BD Biosciences, Franklin Lakes, NJ, USA) as per the manufacturer’s instructions.

### Statistical analysis

Statistical analysis was performed by using GraphPad Prism software version 5.0 (GraphPad software, CA, USA). One way ANOVA was used for comparing lymphocyte proliferative responses and cytokine levels in all 1:5, 1:10 diluted blood and PBMC assays against the antigens and mitogen.

## Results

### IFN-γ secretion in response to MPT51 recombinant antigen of *M. tuberculosis*, PPD and PHA

T lymphocyte function was studied by measuring IFN-γ levels in 1:5, 1:10 diluted WB and PBMCs stimulated with the antigens and mitogen. One way ANOVA was used to compare the differences among the three assay conditions.

**Figure 1:**
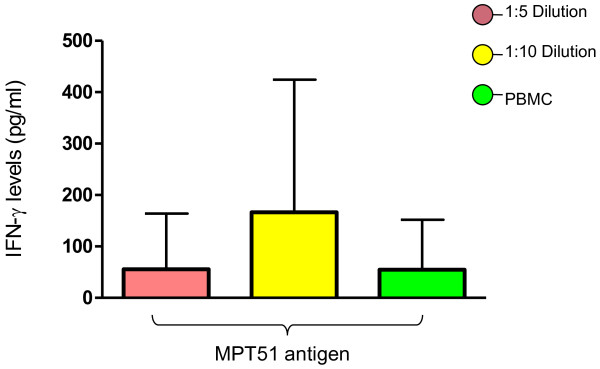
**IFN-γ levels against MPT51 recombinant antigen of *M. tuberculosis.*** Based on the statistical analysis *i.e.*, ONE way ANOVA, there is no significant difference between these assays, and the p = 0.39.

The mean values of IFN-γ levels in 1:5, 1:10 diluted WB and PBMC’s stimulated with MPT 51 recombinant antigen were 55.71pg/ml, 166.3pg/ml and 54.86pg/ml respectively. Though there was a difference in IFN-γ secretion in all the three assay conditions, the difference was not statistically significant (p = 0.39) (Figure 1).

**Figure 2:**
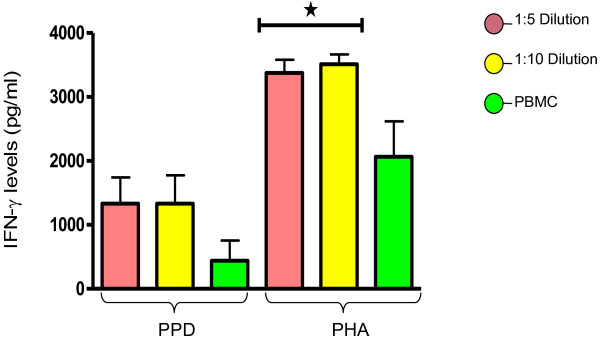
**IFN-γ levels against PPD of *M. tuberculosis *and PHA. **ONE way ANOVA analysis shows that p = 0.20 and 0.0171 for PPD and PHA respectively.

In PPD stimulated 1:5, 1:10 diluted WB and PBMCs cultures, the mean values of secreted IFN-γ levels were 1333pg/ml, 1333pg/ml, and 438.7pg/ml respectively. Here also, the difference was not statistically significant (p = 0.20) (Figure 2).

Whereas the IFN-γ levels in 1:5, 1:10 diluted WB and PBMCs in response to PHA, showed statistically significant difference (p = 0.017) (Figure 2). The mean values of IFN-γ in 1:5, 1:10 diluted WB and PBMC cultures stimulated by PHA were 3375pg/ml, 3513pg/ml and 2064pg/ml respectively.

### Lymphocyte proliferation response against MPT51 recombinant antigen of *M. tuberculosis,* PPD and PHA

Apart from IFN-γ measurement, T lymphocyte function was also studied by its proliferative ability in 1:5, 1:10 diluted WB and PBMC’s stimulated with the antigens and mitogen. The differences among the three assay conditions were compared by using one way ANOVA.

Here, proliferative response of lymphocytes was measured in terms of thymidine incorporation and expressed as stimulation index (SI).

**Figure 3:**
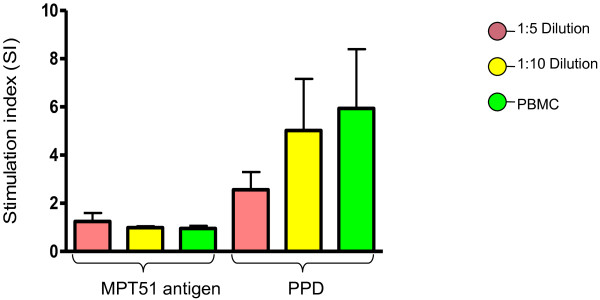
**Lymphocyte proliferation against MPT51 recombinant antigen and PPD of *M. tuberculosis. ***From statistical analysis *i.e.*, ONE way ANOVA, differences between these assays against antigen and PPD are not significant (p = 0.58 and 0.45 respectively).

**Figure 4:**
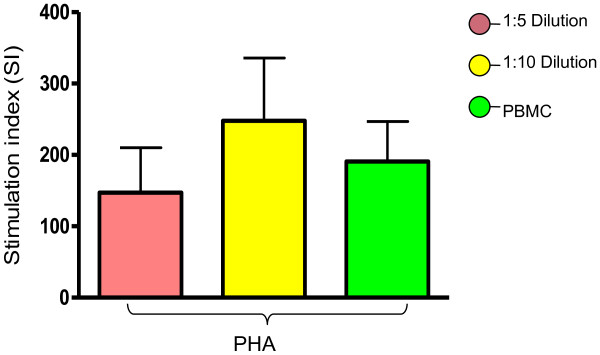
**Lymphocyte proliferation against PHA.** ONE way ANOVA shows p = 0.60. It indicates there is no statistical significance difference between the assays.

In all the assay conditions *i.e.* in 1:5, 1:10 diluted WB and PBMC, lymphocyte proliferation response against MPT 51 recombinant antigen of *M. tuberculosis* was similar (Figure 3). On doing one way ANOVA for comparison, there was no statistically significant difference (*p* = 0.58) among the three conditions. Although, in all the three assay conditions, high thymidine uptake values were observed in response to PPD and PHA (represented positive controls), the difference was not statistically significant (p = 0.45 & 0.60 respectively) (Figure 3 & Figure 4).

These results suggest that the IFN-γ secretion and lymphocyte proliferation patterns were similar in 1:5 and 1:10 diluted WB and in PBMC assays against the antigens and mitogen.

## Discussion

This is a preliminary study, where we included only 8 study subjects to know whether we can use 1:5 and 1:10 diluted whole blood assays or not. Healthy laboratory volunteers were enrolled in this study because their exposure to *M. tuberculosis*, would have made them possess sensitized T cells, which upon re-stimulation with *M. tuberculosis* specific antigens, will induce immune response and secrete cytokines. Since *M. tuberculosis* is an intra cellular pathogen, cell mediated immunity, especially T cells play a crucial role in immune response. T cell function was primarily measured by their proliferative capacity against the crude and recombinant antigens as well as mitogen. Apart from that, IFN-γ is a key cytokine involved in the control of tuberculosis. So, cell proliferation and IFN-γ were used as the parameters to optimize the assay from long term culture condition (6 days). Long-term assays have advantages over short term assays, because during long term incubations, there will be an expansion of antigen specific IFN-γ secreting central memory T- cells. Therefore long-term assays are more sensitive to check diagnostic and vaccine potential of *M. tuberculosis* specific antigens [10].

As described by many authors [11], even though the conventional PBMC assays are specific in T cell functional analysis (since there is no influence of other whole blood components such as neutrophils and plasma), it requires large sample volume and there will be considerable cell loss during isolation.

The alternative T cell assay, WB assay is advantageous over PBMC assays; it requires small sample size, it is rapid and there is no need for cell separation. But the main disadvantage of this technique is that the number of cells cultured is neither known nor controlled. As such, variations in cytokine production between individuals, and in different physiological and pathological states, could represent either changes in the numbers of cytokine producing cells (e.g. T lymphocytes, monocytes) or an altered ability of those cells to produce cytokines, or both. In contrast, purified PBMC cultures include a precisely known number of cytokine-producing cells.

Thus, this study indicates that, although there is substantial inter-individual variation in *ex vivo* cytokine production, the production of any given cytokine by a particular individual is stable in the absence of changes in health and lifestyle factors, and that whole blood cultures can be used instead of purified PBMC cultures to measure a variety of cytokines.

In addition to describing the variation in cytokine production, the aim of this study was to identify whether whole blood cultures could be used to measure cytokine production rather than purified PBMCs. This might be an advantage in field work and/or where large numbers of blood samples need to be processed [12].

In this study, we employed two different whole blood dilution conditions *i.e.* 1:5 and 1:10 dilutions for T cell functional analysis in comparison to PBMC assays. For PBMC cultures, 10% AB serum which has been added provide nutrients that are equal to 1:10 diluted whole blood concentration. Thus, in this study we used the highest dilution factor i.e. 1:10.

Based on one way ANOVA results, we observed no statistically significant difference in lymphocyte proliferation in 1:5, 1:10 diluted WB and PBMC assays against all the three antigens and mitogen.

Whereas in the case of IFN-γ levels, there was no statistically significant difference in all the assays against recombinant protein and crude protein. But there was a significant increase (p = 0.0379) in IFN-γ levels in 1:10 diluted WB assay against mitogen, when compared to PBMC assay.

When compared to 1:5 diluted WB and PBMCs, stimulation index and IFN-γ levels were high in 1:10 diluted WB assays when stimulated with antigens and mitogen. Based on these results we concluded that 1:10 diluted WB assays are more promising and work better than both 1:5 diluted WB and PBMC assays.

In comparison with 1:5 diluted WB and PBMCs, availability of nutrients during long term incubation will be more in 1:10 diluted WB because of higher volume of RPMI 1640 medium, which would have helped to avoid cell crowding during the incubation. This might play an essential role in optimal T-cell activation and secretion of IFN-γ against the antigens and mitogen in 1:10 diluted WB assays.

## Conclusions

Based on these results we conclude that 1:10 diluted WB assays can be used as an alternative immunological assay. When compared to PBMC and 1:5 diluted WB assays, 1:10 diluted WB assays require very small volume of blood, so that these assays are useful in paediatric and field studies. It is also useful in screening large number of antigens in the same blood sample [13]. Thus 1:10 diluted WB assays are more useful than PBMC assays.

## Competing interests

The authors declare that they have no competing interests.

## Authors’ contributions

AD was the primary author of the manuscript. AD had collected blood samples and performed the immunological assays. PM participated in analyzing the data and writing the manuscript. AR designed the study. All authors contributed in reviewing the manuscript. All authors have read and approved the final manuscript.

## References

[B1] World Health Organization Global Tuberculosis Control—Surveillance, Planning, Financing, WHO Report 2011 Geneva: World Health Organization www.who.int/tb/publications/global_report/2011/gtbr11_full.pdf

[B2] Lefford MJ Transfer of adoptive immunity to tuberculosis in mice Infect Immun 1975 11 1174 1181 806520 10.1128/iai.11.6.1174-1181.1975 PMC415196

[B3] Flynn JL Immunology of tuberculosis and implications in vaccine development Tuberculosis (Edinb) 2004 84 93 101 10.1016/j.tube.2003.08.010 14670350

[B4] Flynn JL Chan J Triebold KJ Dalton DK Stewart TA An essential role for interferon gamma in resistance to Mycobacterium tuberculosis infection J Exp Med 1993 178 2249 2254 10.1084/jem.178.6.2249 7504064 PMC2191274

[B5] Ottenhoff TH Kumararatne D Casanova JL Novel human immunodeficiencies reveal the essential role of type-I cytokines in immunity to intracellular bacteria Immunol Today 1998 19 491 494 10.1016/S0167-5699(98)01321-8 9818540

[B6] Hernandez-Pando R Orozcoe H Sampieri A Pavon L Correlation between the kinetics of Thl/Th2 cells and pathology in a murine model of experimental pulmonary tuberculosis Immunology 1996 89 26 33 8911136 PMC1456655

[B7] Cooper AM Dalton DK Stewart TA Griffin JP Russell DG Disseminated tuberculosis in interferon gamma gene-disrupted mice J Exp Med 1993 178 2243 2247 10.1084/jem.178.6.2243 8245795 PMC2191280

[B8] Hanekom WA Dockrell HM Ottenhoff TH Doherty TM Fletcher H Immunological outcomes of new tuberculosis vaccine trials: WHO panel recommendations PLoS Med 2008 5 e145 10.1371/journal.pmed.0050145 18597551 PMC2443198

[B9] Ramalingam B Baulard AR Locht C Narayanan PR Raja A Cloning, expression, and purification of the 27 kDa (MPT51, Rv3803c) protein of Mycobacterium tuberculosis Protein Expr Purif 2004 36 53 60 10.1016/j.pep.2004.01.016 15177284

[B10] Eliane M Leyten S Sandra M Arend Corine Prins Discrepancy between *mycobacterium tuberculosis*-specific gamma interferon release assays using short and prolonged in vitro incubation Clin Vaccine Immunol 2007 14 7 880 885 10.1128/CVI.00132-07 17507543 PMC1951056

[B11] Cruz MIO de Janeiro R Whole blood assay to access T cell-immune responses to mycobacterium tuberculosis antigens in healthy Brazilian individuals Immunology and immunotherapy 2004 99 1 53 55 10.1590/s0074-02762004000100009 15057347

[B12] Yaqoob P Newsholme EA Calder PC Comparison of cytokine production in cultures of whole human blood and purified mononuclear cells Cytokine 1999 11 8 600 605 10.1006/cyto.1998.0471 10433807

[B13] Deenadayalan A Aavudaiyappan Rajendiran A Vaithilingam Velayudham B Frederick S Yang HL Dobos K John T Belisle Raja A Immunoproteomic identification of human T cell antigens of *mycobacterium tuberculosis* that differentiate healthy contacts from tuberculosis patients Mol Cell Proteomics 2009 9 538 549 20031926 10.1074/mcp.M900299-MCP200 PMC2849706

